# Mutations in Intron 1 and Intron 22 Inversion Negative Haemophilia A Patients from Western India

**DOI:** 10.1371/journal.pone.0097337

**Published:** 2014-05-20

**Authors:** Preethi S. Nair, Shrimati D. Shetty, S. Chandrakala, Kanjaksha Ghosh

**Affiliations:** 1 Department of Thrombosis and Haemostasis, National Institute of Immunohaematology (ICMR), Mumbai, Maharashtra, India; 2 Department of Haematology, King Edward Memorial Hospital, Mumbai, Maharashtra, India; National Cerebral and Cardiovascular Center, Japan

## Abstract

Despite increased awareness and diagnostic facilities, 70–80% of the haemophilia A (HA) patients still remain undiagnosed in India. Very little data is available on prevalent mutations in HA from this country. We report fifty mutations in seventy one Indian HA patients, of which twenty were novel. Ten novel missense mutations [*p.Leu11Pro (p.Leu-8Pro), p.Tyr155Ser (p.Tyr136Ser), p.Ile405Thr (p.Ile386Thr), p.Gly582Val (p.Gly563Val) p.Thr696Ile (p.Thr677Ile), p.Tyr737Cys (p.Tyr718Cys), p.Pro1999Arg (p.Pro1980Arg)*, *p.Ser2082Thr (p.Ser2063Thr), p.Leu2197Trp (p.Leu2178Trp), p.Asp2317Glu (p.Asp2298Glu)] two nonsense* [p.Lys396* (p.Lys377*), p.Ser2205* (p.Ser2186*)], one insertion [*p.Glu1268_Asp1269ins (p.Glu1249_Asp1250*)] and seven deletions [*p.Leu882del (p.Leu863del), p.Met701del (p.Met682del*), *p.Leu1223del (p.Leu1204del), p.Trp1961_Tyr1962del (p.Trp1942_Tyr1943del) p.Glu1988del (p.Glu1969del), p.His1841del (p.His1822del), p.Ser2205del (p.Ser2186del)*] were identified. Double mutations (*p.Asp2317Glu; p.Thr696Ile*) were observed in a moderate HA case. Mutations [*p. Arg612Cys (p.Arg593Cys), p.Arg2326Gln (p.Arg2307Gln*)] known to be predisposing to inhibitors to factor VIII (FVIII) were identified in two patients. 4.6% of the cases were found to be cross reacting material positive (CRM+ve). A wide heterogeneity in the nature of mutations was seen in the present study which has been successfully used for carrier detection and antenatal diagnosis in 10 families affected with severe to moderate HA.

## Introduction

Haemophilia A (HA), one of the most common bleeding disorders is caused by defective factor VIII gene (*F8*), leading to deficiency of active factor VIII (FVIIIa). It is one of the largest genes located at Xq28 position at the telomeric end of X- chromosome spanning 186 Kb, comprising of 26 exons and 25 introns encoding 2332 amino acids. A high GC content makes it hypermutable, with approximately 30% of the mutations arising *de novo*. There are 70 CpG dinucleotides within the 9.1 kb coding region of *F8* representing 140 different potential base-pair changes. Human Gene Mutation Database (HGMD) reports a total of 2572 mutations, of which 1514 are missense/nonsense, 168 splicing, 426 small deletions, 140 small insertions, 32 small indels, 228 gross deletions, 38 gross insertions, 19 complex rearrangements, and 7 regulatory mutations [1]. Only two recurrent mutations are reported in *F8* i.e. inversion 1 and 22 which have been reported in 3–5% and 20–50% of severe HA patients in various reports published in literature so far [2], [3].

There are only few reports on the nature of mutations in HA from India [4], [5]. Present study was undertaken to analyse the type of mutations in inversion negative HA patients from Western India and also to offer genetic diagnosis by directly identifying the causative mutations in affected families.

## Materials and Methods

“The study was approved by the Institutional Ethics Committee (Institute of Immunohaematology/Institutional Ethics Committee) IIH/IEC/15–2007. Informed consent duly signed has been taken from patients and all clinical investigation has been conducted according to the principles expressed in the Declaration of Helsinki”.

71 intron 1 and 22 inversion negative cases (24 severe, 22 moderate and 25 mild) attending the Comprehensive Haemophilia Care Centre at National Institute of Immunohematology, Mumbai were included in the study, after taking a detailed clinical history along with pedigree data.

After obtaining informed consent, 9 ml venous blood was collected in 3.2% tri -sodium citrate in the ratio 1∶9 anticoagulant: blood. It was spun at 4000 rpm at 4°C for 15 minutes. The supernatant containing the platelet poor plasma (PPP) was separated and used for phenotypic analysis. The cell pellet was used for DNA extraction which was done by using commercial kits (Invitrogen, CA, USA).

### Phenotypic Assessment

Measurement of the prothrombin time (PT), activated partial thromboplastin time (APTT) and thrombin time (TT) was done using commercial reagents (Dade Behring, Marburg, Germany). Mixing studies at 0 hour, 1 hour and 2 hours were performed in all cases to rule out the presence of inhibitors against FVIII. Factor VIII coagulant activity (FVIII: C) was measured by one-stage assay using commercial deficient plasma (Diagnostica Stago, Asnieres, France) using a semi-automated coagulometer (ST Art, Diagnostica Stago, Asnieres, France). Factor VIII antigen (FVIII: Ag) was assayed by ELISA using commercial kits (Asserachrom FVIII: Ag; Diagnostica Stago, Asnieres, France).

### DNA Analysis

The coding region, intron/exon boundaries and the un-translated regions of the *F8* were amplified in multiplex polymerase chain reactions (MPCR) using specific primers (Sigma Aldrich, Missouri, USA) [5], [6]. These were then screened for mutations using Conformation Sensitive Gel Electrophoresis (CSGE) [7].

The CSGE gel was prepared by using 10% acrylamide (Invitrogen, CA, USA), with 1,4 bis acrolyl piperazine (Fluka, Finland) as a cross linker in the ratio 99∶1, along with mild denaturants 10% ethylene glycol (Sigma Aldrich, Missourie, USA) and 15% formamide (Sigma Aldrich, Missourie, USA). Heteroduplexing was carried out by mixing 4 µl of the DNA amplicon from the patient with 4 µl of the normal PCR product and subjected to heteroduplexing at 98°C for 5 minutes, 65°C for 30 minutes or 98°C for 5 minutes and 55°C for 30 minutes. 4.5 µl of this mixture and 2 µl of gel loading dye were loaded onto the gel, run overnight in a 0.5×Tris- Taurine- EDTA (TTE) buffer. The gel was stained using 0.5-µg/ml ethidium bromide (Promega Corporation, WI, U. S. A). Samples with altered migration profiles were subjected to DNA sequencing (3130 GA sequencer, Applied Biosystems, CA, USA) to confirm the nature of mutation using both forward and reverse primers. Direct DNA sequencing was used to detect mutations in cases where in CSGE did not show mobility shift.

The novel missense mutations were screened in 50 healthy controls to rule out possibility of these being polymorphisms. The novel mutations were verified in HAMSTeRS [8] and HGMD databases [1]. Prediction softwares i.e. SIFT (Sorting Intolerant from Tolerant) [9], PolyPhen (Polymorphism phenotyping) [10], and PANTHER (Protein ANalysis THrough Evolutionary Relationships) [11] were used to predict the deleteriousness of the novel mutations**.** SIFT predicts whether an amino acid substitution affects protein function. SIFT prediction is based on the degree of conservation of amino acid residues in sequence alignments derived from closely related sequences, collected through PSI-BLAST. PANTHER is classification system to classify proteins (and their genes) in order to facilitate high-throughput analysis. PolyPhen performs the prediction through sequence based characterization of the substitution site, calculation of position-specific independent count (PSIC) profile scores for two amino acid variants, and calculation of structural parameters and contacts. The software HOPE (Have yOur Protein Explained) [12] was used to determine the effect of the mutation on the protein. HOPE collects structural information from a series of sources, including calculations on the 3D protein structure, sequence annotations in Uniprot and predictions from Distributed Annotation System servers. HOPE combines this information to analyse the effect of a certain mutation on the protein structure and function.Conservation across the mammalian species were carried out using ClustalW2 alignment software [13].

## Results

Mutations were identified in 62 (5 familial and 57 unrelated) intron 1 and 22 inversion negative congenital HA cases from Western India. 50 individual mutations were observed in the present study with 20 novel mutations. Different types of mutations detected were as follows - 62% (n = 31/50) missense, 8% (n = 4/50) insertion, 12% (n = 6/50) nonsense, 16% (n = 8/50) deletions and 2% (n = 1/50) splice site. 60% (n = 30/50) of the mutations were detected in the A domain. 22 out of 31 (70.6%) missense mutations were in the A domain, followed by 25.8% (n = 8/31) in C domain. 7 out of 12 (58.3%) frame shift mutations were located in the B domain. In 53 patients, mutations were picked up by CSGE followed by DNA sequencing, while in the remaining 12 patients, mutations were detected by direct DNA sequencing. In 8 cases mutations were undetected despite sequencing the entire gene.

Three patients (ID 61, 62 and 68) had a missense mutation i. e. *p.Arg550Cys (p.Arg531Cys)* out of which two (ID 62 and 68) were found to be CRM reduced (CRM _red_). Patient (ID 1) with moderate deficiency of FVIII had a possible inhibitor predisposing mutation i.e. *p.Arg612Cys (p.Arg593Cys)* in exon 12 corresponding to A2 domain. This mutation has been observed to be mildly predisposing towards inhibitor formation especially in presence of peri-operative use of FVIII [14], [15]. However this patient was not extensively transfused till the date of blood sampling. Another patient (ID 104) was found to carry two mutations i.e. *p.Asp2317Glu (p.Asp2298Glu) and p.Thr696Ile (p.Thr677Ile)* in C and A domains respectively, both of which were novel (Table 1).

**Table 1:** Mutations identified in the present study.

Pt ID	Clinical Manifestation	Age at presentation (yrs)	FVIII:C (%)	FVIII: Ag (%)	Transfusion product	Inhibitor status	Exon	Nucleotide position	*Amino acid change (HABD)*	*Amino acid change (HGVS)*	Functional Domain	CRM status	Novel/Reported
100	Mild	62	7.9	10	FVIII, Haemostat	Neg	1	c.32T>C	*p.Leu-8Pro*	*p.Leu11Pro*	Signal Peptide	Neg	**Novel**
102	@ Moderate	8	2.2	<1	FFP, Cryo	Neg	4	c.464A>C	*p.Tyr136Ser*	*p.Tyr155Ser*	A1	Neg	**Novel**
103	@ Moderate	6	3.3	17	FVIII	Neg	4	c.464A>C	*p. Tyr136Ser*	*p. Tyr155Ser*	A1	Neg	**Novel**
20	Severe	NA	<1	<1	Never	Neg	7	c.857A>C	*p. His267Pro*	*p. His286Pro*	A1	Neg	Reported
6	Mild	3	18	25	ND	Neg	7	c.883T>C	*p. Phe276Leu*	*p. Phe295Leu*	A1	Neg	Reported
106	Mild	27	22	30	Cryo	Neg	7	c.923C>T	*p.Ser289Leu*	*p.Ser308Leu*	A2	Neg	Reported
94	Moderate	1	4.2	<1	ND	Neg	8	c.1214T>C	*p.Ile386Thr*	*p.Ile405Thr*	A2	Neg	**Novel**
77	Mild	20	40	42	Whole Blood	Neg	9	c.1337G>A	*p.Arg427Gln*	*p.Arg446Gln*	A2	Neg	Reported
19	Mild	5.5	7.4	10	Never	Neg	10	c.1475A>G	*p.Tyr473Cys*	*p.Tyr492Cys*	A2	Neg	Reported
101	Moderate	1.5	2	2.4	Never	Neg	10	c.1475A>G	*p.Tyr473Cys*	*p.Tyr473Cys*	A2	Neg	Reported
11	Moderate	50	3.2	20	Whole Blood, FFP	Neg	10	c.1492G>A	*p.Gly479Arg*	*p.Gly498Arg*	A2	Neg	Reported
44	Mild	NA	6	15	Never	Neg	10	c.1492G>A	*p.Gly479Arg*	*p.Gly498Arg*	A2	Neg	Reported
51	Mild	28	17	<1	Cryo	Neg	10	c.1492G>A	*p.Gly479Arg*	*p.Gly498Arg*	A2	Neg	Reported
61	Moderate	18	2.8	11	Never	Neg	11	c.1648C>T	*p.Arg531Cys*	*p.Arg550Cys*	A2	Neg	Reported
62	Moderate	7	5	27	FFP, Cryo	Neg	11	c.1648C>T	*p.Arg531Cys*	*p.Arg550Cys*	A2	Redu	Reported
68	Mild	38	12	46	Never	Neg	11	c.1648C>T	*p.Arg531Cys*	*p.Arg550Cys*	A2	Redu	Reported
29	Moderate	14	3	210	Never	Neg	11	c.1745G>T	*p.Gly563Val*	*p.Gly582Val*	A2	Pos	**Novel**
1	Moderate	20	1.5	2.8	Cryo	Neg	12	c.1834C>T	*p.Arg593Cys*	*p.Arg612Cys*	A2	Neg	Reported
45	Mild	16	40	50	Never	Neg	14A	c.2149C>T	*p.Arg698Trp*	*p.Arg717Trp*	A2	Neg	Reported
93	Mild	18	16	5.5	Never	Neg	14A	c.2167G>A	*p.Ala704Thr*	*p.Ala723Thr*	A2	Neg	Reported
71	Mild	8	29	60	Never	Neg	14A	c.2210A>G	*p.Tyr718Cys*	*p.Tyr737Cys*	A2	Redu	**Novel**
86	Mild	NA	8.5	200	Never	Neg	14K	c.5122G>A	*p.Arg1689Cys*	*p.Arg1708Cys*	A3	Pos	Reported
14	Moderate	NA	4.6	8.5	Never	Neg	16	c.5398C>T	*p.Arg1762Cys*	*p.Arg1781Cys*	A3	Neg	Reported
105	Moderate	8	4.3	6	Whole Blood	Neg	16	c.5399G>A	*p.Arg1762His*	*p.Arg1781His*	A3	Neg	Reported
97	Mild	12	13	80	Whole Blood	Neg	16	c.5526G>A	*p.Met1823Ile*	*p.Met1842Ile*	A3	Pos	Reported
54	# Severe	49	<1	<1	FVIII	Neg	16	c.5573C>G	*p.Ser1839Cys*	*p.Ser1858Cys*	A3	Neg	Reported
55	# Moderate (Female HA)	16	2	NA	Never	Neg	16	c.5573C>G	*p.Ser1839Cys*	*p.Ser1858Cys*	A3	Neg	Reported
99	Mild	16	10	16	FVIII, Cryo	Neg	18	c.5879G>A	*p.Arg1941Gln*	*p.Arg1960Gln*	A3	Neg	Reported
28	Mild	15	15	20	Never	Neg	18	c.5996C>G	*p.Pro1980Arg*	*p.Pro1999Arg*	A3	Neg	**Novel**
38	Mild	6	42	17	Never	Neg	21	c.6245G>C	*p.Ile2061Asn*	*p.Ile2080Asn*	C1	Neg	**Novel**
108	Mild	60	11.5	28	Never	Neg	22	c.6296T>A	*p.Ser2063Thr*	*p.Ser2082Thr*	C1	Neg	Reported
79	Severe	NA	<1	<1	ND	Neg	23	c.6544C>T	*p.Arg2163Cys*	*p.Arg2182Cys*	C1	Neg	Reported
17	Moderate	7.5	4.2	6.7	Never	Neg	23	c.6545G>A	*p.Arg2163His*	*p.Arg2182His*	C1	Neg	Reported
69	Moderate	14	4.6	7.5	Whole Blood	Neg	24	c.6590T>G	*p.Leu2179Trp*	*p.Leu2197Trp*	C2	Neg	**Novel**
64	Severe	NA	<1	<1	ND	Neg	24	c.6683G>A	*p.Arg2209Gln*	*p.Arg2228Gln*	C2	Neg	Reported
72	*Severe	20	<1	<1	Packed cells	Neg	24	c.6683G>A	*p.Arg2209Gln*	*p.Arg2228Gln*	C2	Neg	Reported
73	*Severe	30	<1	<1	Packed cells	Neg	24	c.6683G>A	*p.Arg2209Gln*	*p.Arg2228Gln*	C2	Neg	Reported
104	Moderate	7	3	42	FFP	Neg	26	c.6951C>G	*p.Asp2298Glu*	*p.Asp2317Glu*	C2	Redu	**Novel**
							13	c.2087C>T	*p.Thr677Ile*	*p.Thr696Ile*	A2		**Novel**
75	Mild	3	14	<1	Never	Neg	26	c.6977G>A	*p.Arg2307Gln*	*p.Arg2326Gln*	C2	Neg	**Novel**
34	Severe	3	<1	<1	ND	Neg	8	c.1186A>T	p.Lys377*	p.Lys396*	A2	Neg	**Novel**
42	$ Severe	20	<1	<1	Whole Blood	Neg	13	c.1965C>G	p.Tyr636*	p.Tyr655*	A2	Neg	Reported
43	$ Severe	20	<1	<1	Whole Blood	Neg	13	c.1965C>G	p.Tyr636*	p.Tyr655*	A2	Neg	Reported
30	Moderate	5	4	2	Never	Neg	16	c.5561G>A	p.Thr1835*	p.Thr1854*	A3	Neg	Reported
74	Moderate	30	1.1	2	Whole Blood	Neg	23	c.6496C>T	p.Arg2147*	p.Arg2166*	C1	Neg	Reported
82	Severe	6	<1	<1	Never	Neg	24	c.6614C>A	p.Ser2186*	p.Ser2205*	C2	Neg	**Novel**
50	Mild	12	26	40	Never	Neg	26	c.6977G>T	p.Arg2307*	p.Arg2326*	C2	Neg	Reported
60	Mild	45	5.2	1.25	Never	Neg	26	c.6977G>T	p.Arg2307*	p.Arg2326*	C2	Neg	Reported
2	Mild	4	7	10	Never	Neg	13	c.2102delT	*p.Met682del*	*p.Met701del*	A2	Neg	**Novel**
52	Severe	12	<1	<1	Never	Pos	14B	c.2645delT	*p.Leu863del*	*p.Leu882del*	B	Neg	**Novel**
76	Moderate	35	<1	<1	FFP	Neg	14E	c.3668delT	*p.Leu1204del*	*p.Leu1223del*	B	Neg	**Novel**
87	Moderate	21	2.6	<1	Never	Neg	14G	c.4379delA	*p.Asn1441Ile fsX5*	*p.Asn1460Ile fsX5*	B	Neg	Reported
35	Severe	18	<1	<1	Never	Neg	16	c.5521_5523 del CAT	*p.His1822del*	*p.His1841del*	A3	Neg	**Novel**
36	Severe	30	<1	<1	FFP, Cryo	Neg	18	c.5883_5884 del GT	*p.Trp1942_ Tyr1943del*	*p.Trp19461_ Tyr1962del*	A3	Neg	**Novel**
65	Severe	13	<1	<1	ND	Neg	18	c.5963_5964 del AG	*p.Glu1969del*	*p.Glu1988del*	A3	Neg	**Novel**
88	Moderate	11	1.5	3.7	FFP	Neg	24	c.6615del A	*p.Ser2186del*	*p.Ser2205del*	C2	Neg	**Novel**
15	∧Severe	3	<1	<1	Never	Neg	14C	c.2945 dupA	*p.Asn963Lys fsX9*	*p.Asn982Lys fsX9*	B	Neg	Reported
16	∧Severe	8	<1	<1	Never	Neg	14C	c.2945 dupA	*p.Asn963Lys fsX9*	*p.Asn982Lys fsX9*	B	Neg	Reported
85	Mild	32	8.5	11.5	Never	Neg	14E	c.3804 dupA	*p.Glu1249_Asp1250 ins*	*p.Glu1268_Asp1269 ins*	B	Neg	**Novel**
18	Severe	11	<1	<1	FFP	Neg	14G	c.4379 dup A	*p.Asn1441LysfsX1*	*p.Asn1460LysfsX1*	B	Neg	Reported
78	Severe	NA	<1	<1	Never	Neg	14G	c.4379 dup A	*p.Asn1441LysfsX1*	*p.Asn1460LysfsX1*	B	Neg	Reported
92	Severe	NA	<1	<1	Never	Neg	14J	c.4825 dup A	*p.Thr1590AsnfsX3*	*p.Thr1609AsnfsX3*	B	NA	Reported
95	Moderate	2.6	<1	<1	Never	Neg	IVS5 Donor Splice Site	c.670G>T	*p.Gly205Trp*	*p.Gly224Trp*	A1	Neg	Reported

*Neg- Negative, Pos- Positive, Redu-Reduced, ND- No details, FFP-Fresh Frozen Plasma, Cryo-Cryoprecipitate.*

All the 10 novel missense changes were analysed in 50 healthy volunteers and none of them were found to carry these changes in addition to the 73 patients analysed in the present study. CSGE was found to be 71.9% (41/57 unrelated HA cases) sensitive in picking up the point mutations, smaller deletions and insertions. The deleteriousness of the novel missense mutations was checked using various prediction softwares (Table 2). 3/62(4.8%) of the cases were found to be CRM+ve with 1 having moderate and 2 having mild clinical manifestations (Table 1). The previously unidentified mutations (novel) were found to be conserved across the species (Tab 2).

**Table 2:** Prediction of the nature of novel missense mutations in A and C domain.

Mutation	SIFT (score-0.0)	PolyPhen (Score)	PANTHER (score)	Possible effects of this mutation HOPE(Have yOur Protein Explained)	Conservation across species (H/M/R/D/O)
*p.Leu11Pro(Leu-8Pro)*	Intolerant	Probably damaging (0.984)	Deleterious (0.5879)	Hydrophobic →hydrophilic, can affect protein folding	L/L/L/L/L
*p.Tyr155Ser(Tyr136Ser)*	Intolerant	Probably damaging (1.0)	Deleterious (0.59947)	Metal ion interaction affected	Y/Y/Y/Y/Y
*p.Ile405Thr(Ile386Thr)*	Intolerant	Probably damaging (0.999)	Deleterious (0.99218)	Threonine (smaller)- loss of interactions, hydrophobicity changes- affecting core or surface protein interactions, Change from Non-polar amino acid to polar, can affect protein stability	I/I/I/I/I
*p.Gly582Val(Gly563Val)*	Intolerant	Probably damaging (1.0)	Deleterious (0.70955)	Glycine(most flexible, buried residue) → Valine (larger size) can disrupt the backbone and thus protein folding	G/G/G/G/G
*p.Thr696Ile(Thr677Ile)* *	Intolerant	Probably damaging (1.0)	Deleterious (0.50928)	Isoleucine(bigger, more hydrophobic) - can affect hydrogen bonds and/or disturb correct folding	T/T/T/T/T
*p.Tyr737Cys (Tyr718Cys)*	Intolerant	Probably damaging (0.998)	Deleterious (0.5076)	Cysteine (smaller) - loss of interaction, more hydrophobic- can disrupt hydrogen bonds and/or affect correct folding	Y/Y/Y/Y/Y
*p.Pro1999Arg(Pro1980Arg)*	Intolerant	Probably damaging (1.0)	Deleterious (0.94734)	Change in polarity- repulsion of ligands, disruption of local conformation by affecting the backbone, loss of hydrophobic interactions either in the core or surface protein, bigger size or Arginine can lead to bumps	P/P/P/P/P
*p.Ser2082Thr(Ser2063Thr)*	Intolerant	Probably damaging (0.989)	Benign (0.28791)	Threonine (bigger) - can lead to bumps,Phosphorylation could affect protein signalling	S/S/S/S/S
*p.Leu2197Trp(Leu2178Trp)*	Intolerant	Probably damaging (1.0)	Deleterious (0.95649)	Hydrophobic→hydrophilic, can destabilize protein, Tryptophan (bigger) can lead to bumps	L/L/L/L/L
*p.Asp2317Glu(Asp2298Glu)* *	Intolerant	Benign (0.005)	Benign (0.08274)	Glutamate (bigger) – leads to bumps; can affect multimeric interactions, has additional methylene group, can form tight binding site for Calcium	N/N/N/N/N

*Double mutation identified in the same patient.

H- Homo sapiens, M- Mus musculus, R-Rattus norvegicus, D-Dasypus novemcinctus, O- Oryctolagus cuniculus.

### Genetic Diagnosis

Genetic diagnosis was successfully offered in 10 families (3 antenatal, twice in the same family and 8 carrier- families) (Table 3).

**Table 3:** Genetic diagnoses successfully offered using the present study.

Sr.No	Mutation in proband	Diagnosis
1	Ex 9; c.1315G>A, p.*Gly439Ser (p.Gly420Ser*	Heterozygous niece, Homozygous Sister (Female HA)
2	Ex 21; c.6226G>T, p.Gly2076* (p.Gly2057*)	First Foetus Affected, Second Foetus Unaffected
3	Ex 23; c. 6545G>A, *p.Arg2182Cys (p.Arg2163Cys)*	Carrier sister 1, Non carrier sister 2
4	Ex 16; c. 5573C>G, *p.Ser1858Cys (p.Ser1839Cys)*	Carrier Daughter- Carrier with FVIII:C levels- 3%
5	Ex 24; c.6683G>A, *p.Arg2228Gln (.Arg2209Gln)p*	Foetus unaffected
6	Ex 14J; c.4819_4825dupA *p.Thr1609AsnfsX3 (p.Thr1590AsnfsX3)*	Foetus unaffected
7	IVS 4;c.388+2GAGTdel	Sister Non-carrier
8	Ex 23; c.6544C>T, *p.Arg2182Cys (p.Arg2163Cys)*	Foetus Unaffected
9	Ex 8; c.1186A>T, p.Lys396* (p.Lys377*)	Sister Non-carrier, Aunt 1-Carrier, Aunt 2- Carrier
10	Ex 16; c.5521_5523delCAT*p.His1841del (p.His1822del)*	Sister-Carrier

## Discussion and Conclusion

There are currently about 16000 registered HA cases with Haemophilia Federation India (HFI). As per the general prevalence of 1 in 5000 male births, population of the country and male: female ratio in the country, India should have more than 125,000 severe haemophilia cases. Thus there is a gross unawareness and under-diagnosis of this disease in the country. Very few laboratories are currently performing genetic diagnosis for haemophilia using different techniques which include indirect linkage technique, CSGE, dHPLC and direct DNA sequencing. There are only two reports earlier on nature of the mutations in HA from North and South of India [4], [5]. The major objective of this study was thus to complement already existing data with the mutational spectrum from Western India besides its utility in carrier detection and antenatal diagnosis in affected families.

It is important to note that, not only certain alterations but also the position of those changes in the 3D structure of the protein plays a critical role in deciding the phenotype of the patient. Thus many- a- times, the same kind of mutation can give rise to different phenotypes [8]. A varied nature of mutations has been observed worldwide in HA patients, due to the highly heterogeneous nature of *F8* gene, its complexity, size and GC rich content. In the current study, for e.g, though there are 4 arginine to cysteine changes, all occur at different residues in FVIII, causing varied degree of clinical manifestations.

All the novel mutations in this series were found to be in the conserved region across the species and these were found to be in conserved regions.

### Missense Mutations

#### Domain A

In a family with patients (ID 102,103), having moderate FVIII deficiency a missense mutation i.e. *p.Tyr155Ser (p.Tyr136Ser)* was detected in the A1 domain. Tyrosine contains a phenol group whereas Serine has hydroxyl group, thus catering to different roles. Tyrosine is involved in metal –ion contact (Ca2+) and the fact that serine is a smaller residue that tyrosine can hamper this function [12]. Patient (ID- 94) having moderate FVIII deficiency showed a missense mutation at amino acid position 405 in the A2 domain, wherein Isoleucine was replaced by threonine *(p.Ile386Thr)*. Isoleucine (a nonpolar-neutral essential amino acid) is one of the 3 amino acids having branched hydrocarbon side chains and is usually interchangeable with leucine and occasionally with valine. The side chains of these amino acids are not reactive and therefore not involved in any covalent chemistry in enzyme active centres. However, these residues are critically important for ligand binding to proteins, and play a central role in protein stability. β carbon of isoleucine is also optically active, just as the β carbon of threonine. The wild type residue i.e. isoleucine is more hydrophobic in nature compared to threonine; in such a case the hydrophobic interaction either in the core or the surface of the protein will be lost. Also threonine is smaller residue as compared to isoleucine, which can result in loss in interactions [12]. The other changes reported at the same residue are either serine, which is small in size compared to isoleucine and polar in nature or phenylalanine [15].

A patient (ID 29) showed a mutation in exon 11; i.e. *p.Gly582Val (p.Gly563Val)* which was detected by direct sequencing technique. Herein G→A change was noted at nucleotide position 1745, wherein glycine at amino acid position 582 was replaced by valine. Introduction of a valine in place of glycine has the potential to seriously disrupt the localized folding in the protein. This is so because glycine is the most flexible amino acid and a change to valine which is a larger residue can affect the torsion angles and force the local backbone into an incorrect conformation and distract the local structure [12]. Subtle alterations of the native conformation could be sufficient to disrupt transport out of the endoplasmic reticulum [16]. Besides, this residue is located in the buried region sensitive to mutation within the A2 domain, thus can cause disruption in the A2 domain structure.

Mutations at or after critical arginine residues at thrombin cleavage sites [residues 391/392 (372/373) and 1708/1709 (1689/1690)] have been shown to render molecule resistant to thrombin activation thus resulting in reduced coagulant activity. Thrombin cleavage site at residue 1708 showed a change from arginine to cysteine in a mild HA patient (ID 86).

A missense mutation was detected in patient (ID 28) with mild FVIII deficiency, wherein the amino acid proline at position 1999 was replaced by arginine (*p.Pro1980Arg)*, in the A3 domain. The mutated residue is involved in multimer contact. The mutation introduces a bigger residue at this position; this can disturb the multimeric interactions. The mutation introduces a less hydrophobic residue. Sometimes, hydrophobicity is important for multimerisation and therefore this mutation could affect the multimer contacts. Prolines are known to be very rigid and therefore induce a special backbone conformation which might be required at this position. The mutation can disturb this special conformation disrupting local structure. The wild type residue has a neutral charge whereas, the mutation introduces a positive charge; this can cause repulsion of ligands or other residues with the same charge. Hydrophobic interactions, either in the core of the protein or on the surface, will be lost [12]. The other change observed at this position was a proline to leucine change in a mild HA case [8].

#### Domain C

A patient (ID 64) was observed to harbour a reported mutation *p.Arg2228Gln (p.Arg2209Gln)* in the region important for VWF binding to FVIII. Failure of vWF binding causes instability to FVIII and thus severe manifestation. Two hotspot mutations *p.Arg2182His (p.Arg2163His)* and *p.Arg2182Cys (p.Arg2163Cys)* were also identified in a case with moderate deficiency of FVIII (ID 17) and another case with severe FVIII deficiency (ID 79).

### Protein Truncating Mutations

#### Domain A

A novel nonsense mutation i.e. c.1186 A>T, p.Lys396* (p.Lys377*) was identified in a severe case; a missense mutation at the same position i.e. *p.Lys396Met (p.Lys377Met)* has been reported earlier [17].

#### Domain C

A protein truncation was observed in two unrelated mild HA cases (ID 50, 60), at amino acid position Arg2326 *(p.Arg2307)*, which is in the region involved in phospholipid binding. The other changes observed at the same position are arginine to leucine [8] predominantly giving rise to severe phenotype, proline, whereas a change to glutamine has been noted in mild/moderate cases. Although in most cases a protein truncation gives rise to a severe clinical manifestation, a moderate case with this same change was reported in the HADB database [8], [18].

### Deletions and Insertions

#### Domain B

Two novel changes *p.Glu1268_ Asp1269 ins (p. Glu 1249_Asp1250)* in a mild patient (ID 85), *p.Leu1223del (p.Leu1204del)* in a moderate patient (ID 76) were detected. Small deletions in B domain have been observed to give rise to milder forms of HA [19]. The residue Asn1460 is reported to be hypermutable with both insertion i.e. *p.Asn1460LysfsX1 (p. Asn1441LysfsX1)* as well as deletion i.e. *p.Asn1460IlefsX5 (p. Asn1441IlefsX5)*, multiple times in the mutation database [1]. In line with these observations, *p.Asn1460LysfsX1 (p. Asn1441LysfsX1)* was identified in two severe patients (ID 18, 78), whereas *p.Asn1460IlefsX5 (Asn1441IlefsX5)* was detected in a case with moderate HA patient (ID 87). Both the above mentioned changes, although have been observed mainly giving rise to severe manifestations, HADB reports certain moderate cases harbouring these changes. The restoration of the reading frame by polymerase errors or ribosomal frame shift may occur in mild/moderate cases thus explaining the less severe phenotype.

#### Domain C

Different substitutions at amino acid Ser2205 were observed to result in different degrees of severity. p.Ser2205*(p. Ser2186*) was detected in a patient with severe FVIII deficiency (ID 82) and *p.Ser2205del (p. Ser2186del) was associated with* moderate deficiency of FVIII in another patient (ID 88).

A domain was observed to harbour maximum number of mutations, followed by the C domain, which is in line with earlier reports [19], [20]. The B domain of *F8* is encoded by exon 14 spanning residues 760–1667(741–17648). This domain also contains 19 of the 25 aspargine (N-) linked glycosylation attachment sites throughout the FVIII molecule. Although the B domain is not directly involved in the central procoagulant activity of FVIII, recent reports have enlightened that B domain participates in intracellular interactions, regulating quality control, secretion and also has potential regulatory roles within plasma during activation, platelet binding, inactivation and clearance [21], [22]. C domain is a functionally significant domain of FVIIII and participates in multiple functions. The residues 2200–2262 (2181–2243) and 2322–2351(2303–2332) are involved with vWF and phospholipid binding respectively. Thrombin binding site is between residues 2262–2267(2243–2248), whereas residues 2267–2304 are involved in releasing vWF and residues 2272–2289 (2253–2270) take part in binding FXa.

### CRM and Mutations

We detected 2 CRM reduced (CRM_red_) cases (ID 62, 68) harbouring *p.Arg550Cys (p.Arg531Cys)*, a hotspot mutation associated with CRM positive cases in earlier reports [19], [23]. Both these patients had mild to moderate deficiency of FVIII. Another case (ID 61) with the same mutation was found to be CRM negative. This change in CpG region has been reported several times in the HAMSTeRS database as occurring in mild/moderate HA patients and was also identified in other Indian studies [5], [24], suggesting that this change predominantly does not cause severe clinical manifestation in HA patients. Another case with mild deficiency of FVIII had *p.Tyr737Cys (p.Tyr718Cys)* mutation (ID 71) and was found to be CRM_red_, but not reported earlier in database. Tyrosine (polar) which is an important residue for post –translational modification of the protein by sulfation was replaced by cysteine (non-polar). The wild type residue tyrosine at position 737(718) was changed to cysteine in a mild HA {ID-71) case. Cysteine is smaller in size and more hydrophobic in nature as compared to tyrosine. The change introduces a more hydrophobic residue which can result in loss of hydrogen bonds and/or disturb correct protein folding [12]. 2 cases with discordant FVIII levels were noted to have a glycine to valine change at different residues i.e. 582, 164.

### Inhibitors and Mutations

Mutation *p.Arg612Cys (p.Arg593Cys)* in exon 12 located in the A2 domain has been reported to be associated with CRM reduced state [14], as well as exhibiting an increased risk factor for inhibitor formation [25]. It was proposed that the intracellular accumulation and subsequent degradation could be responsible for lower FVIII activity and antigen in mild HA patients with this change, thus causing a CRM reduced state. We detected this change in one of the patients (ID 1) in the present study. Herein, arginine, (–NH2 group), located in the superficial pocket is changed to cysteine (a–SH group). This change is located in a residue located in helix I, conserved only in the aligned FVIII protein. Arginine has a guanidine group, which ensures that is always positively charged. On the other hand, the cysteine residue can be easily oxidised at its -SH group. The new charge distribution, induced by the mutation, could alter the protein functions. This mutation has also been found in moderate and mild HA forms reported in the *F8* mutation database. A combination of peri-operative use of FVIII, especially administered by a continuous inflow and mutation *p.Arg612Cys (p.Arg593Cys)* posed a higher risk towards forming FVIII inhibitors [25]. However, the patient in the present study was transfused only with 6000 IU of FVIII on 2 occasions, till the time of blood sampling.

Desmopressin (1-deamino-8-D-arginine vasopressin) is a prefered first line of treatment for mild/moderate HA patients due to its cost effectiveness compared to FVIII concentrates and safety due to absence of viral transmissions. There is a three to five fold increase in FVIII concentration in vivo, but individual responses vary. Factors such as age, baseline FVIII: C activity has been observed to be predictive desmopressin-response conditions, but it has been observed that certain mutations like *Arg2169His (Arg2150His) and Pro149Arg (Pro130Arg)*, show no change in their depleted FVIII: C levels in spite of desmopressin treatment. The change *p.Arg612Cys (p.Arg593Cys)* was found to be exhibiting a 100% initial response to desmopressin treatment and even after 6 hours retained 80% of this effect [26]. The patient in this study can be treated with desmopressin as the first line of treatment.

Missense mutations in the light chain are more often (12%) associated with inhibitors. In the C2 domain, with mutations clustered in and around residues 2257–2331 (2238–2312) tend to be associated with high risk in developing inhibitors. Such mutations may give rise to conformational changes in patient’s FVIII molecule which would become antigenically distinct from wild type molecule. The change *p.Arg2326Gln (p.Arg2307Gln)* detected in a mild case (ID 75) resides in the above mentioned cluster of residues. This change has been noted quite frequently in the HAMSTeRS database, reiterating its importance in the FVIII stability. The patient in the present study was only 3 years of age and was transfused only once with FFP till the time of sampling. In the wake of lesser exposures to FVIII transfusions, a future follow up study in these cases will confirm the association of this mutation with inhibitors; meanwhile the patients were duly counselled to undergo regular inhibitor tests.

Furthermore, though it has been reported that missense mutations pose lesser risk for inhibitor formation, the kind of amino acid change i.e. substitutions belonging to different physicochemical class than the original residue can increase the risk of inhibitor formation [27]. The changes *p.Ala723Thr(p.Ala704Thr)* ID-93, *p.Arg2182His(p.Arg2182His)* ID-79, *p.Arg2228Gln(p.Arg2209Gln)* ID- 64, 72, 73 and *p.Arg2326Gln(p. Arg2307Gln)* ID- 75 other than the previously mentioned mutations, can pose a threat towards inhibitor formation due to their vast physico-chemical property changes [27].

### Double Mutations

A serious concern about the presence of multiple mutations is the possibility of misdiagnosis in genetic diagnosis of affected families. In many parts of the world, once the causative mutation is identified, screening of the rest of the gene is stalled. We have earlier reported presence of 7.2% (7 out of 97 unrelated HB cases) double mutations in case of *F9* [28], [29]. We have earlier reported an incidence of a familial case with a double mutation, in which one of the missense mutations was novel *p.His330Arg (p.His311Arg),* whereas the concomitant change in a hypermutable site i.e. *p.Arg2228Gln (p.Arg2209Gln)* reported earlier [23] was detected. Another moderate HA case (ID 104) i.e. *p.Thr696Ile (p.Thr677Ile)* in exon 13 and *p.Asp2317Glu (p.Asp2298Glu) in exon 26* was detected. Glutamate has additional methylene group and can form tight binding site for Calcium. Whenever a missense mutation is encountered, it is a common practice to know the nature of the mutation either by prediction softwares or screen for the same change in normal healthy controls. Though both these novel missense mutations were neither detected in other patients nor in normal healthy controls, the possibility of one of them being a benign change cannot be ruled out. In vitro gene expression studies will confirm the effect of such novel changes in the protein; however many of the laboratories will not have an access to these facilities.

In the present series, in 11 cases, mutations could not be identified. This has been reported by several workers earlier, wherein mutations have not been detected in 2–10% of the HA cases, even after directly sequencing the coding, splice site and the promoter region of *F8* [30], [31]. Deep intronic mutations, epigenetic factors are some of the possible explanations which need further evaluation. CSGE was found to be 72% sensitive in the current study; it can be used as an initial screening technique for the identification of mutations, wherein cost is a constraint.

Although descriptive in nature, this study tries to emphasize the high heterogeneity of the *F8* gene and the fact that the nature and the position of the change can influence the degree of severity of HA. There is a need for such a data from a hugely populated country like India for enabling better genetic diagnoses to affected families and in turn create more awareness of this under-diagnosed disorder. The study also reiterates the need to analyse the entire *F8,* to identify all the variations in order to offer an efficient genetic diagnoses. Even when the *F8* mutations stand a chance to influence the phenotype variability of severe HA cases [32], it is not the sole player in determining the bleeding patterns and determining the treatment decisions [33]. Various other genetic and environmental factors can influence the course of events in a HA patient’s clinical manifestations thereby affecting the treatment regimen. Nevertheless, studying the mutations can help in identifying causative mutations, offer genetic diagnoses to affected families and detect certain inhibitor predisposing mutations, which can thus influence the treatment process.
